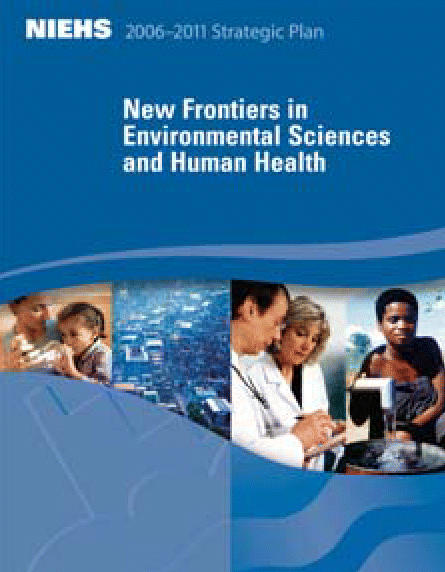# A Hitchhiker’s Guide to the NIEHS Strategic Plan

**DOI:** 10.1289/ehp.114-a334

**Published:** 2006-06

**Authors:** David A. Schwartz

**Affiliations:** Director, NIEHS and NTP E-mail: david.schwartz@niehs.nih.gov

In the lexicon of organizations, government ones especially, the words “strategic plan” may often be perceived as a synonym for “long, boring, inscrutable document full of jargon.” Not so the NIEHS Strategic Plan. Conceived as a means of structuring and communicating the scientific goals of the institute, it is, by design, a concise yet descriptive tool that will continue to evolve and change to meet its purpose. Although the complete Strategic Plan provides a thorough overview of the NIEHS goals and challenges (which I encourage you to explore), what follows is a quick tour of the plan’s history and highlights—a “hitchhiker’s guide,” if you will.

The NIEHS Strategic Plan, distributed as a supplement to the May issue of EHP and available online, is the result of a nearly year-long process of discussions with more than 400 scientific and public leaders from academia, government, medical professions, community advocacy groups, and the general public. It was drafted with input from a national web survey, the participation of 90 individuals during a two-day Strategic Planning Forum, discussions with members of the NIEHS Public Interest Liaison Group, numerous opportunities for public review and comment on draft documents, and much input from NIEHS staff and members of the NIEHS National Advisory Environmental Health Sciences Council. However, now that the Strategic Plan has been released, there are two obvious next questions: what does it mean, and how will it impact the future opportunities and direction of our field?

While our vision is quite broad—to prevent disease and improve human health by using environmental sciences to understand human biology and human disease—putting this vision into practice will require that we focus our attention on understanding all aspects of the etiology and pathogenesis of complex human diseases. Our view is that we have, with the Strategic Plan, created a matrix of challenges and goals that will guide NIEHS development over the next several years, and dovetail our efforts with those outlined in the NIH Roadmap to accelerate the application of our science to improvements in human health and disease. For each of the plan’s seven goals in the areas of clinical research, basic research, integrative research programs, community-linked research, biomarker development, training, and partnerships, the challenge will be to define the programmatic scope, assess the ability to integrate science across disciplines, and identify the potential impact of this research on public health.

The NIEHS has already begun implementing new programs to meet the goals and objectives of the Strategic Plan. Allow me to point out some of the highlights. In the area of integrative research programs, the NIEHS has developed a new Office of Translational Research and recruited Dr. William Martin to direct it. We have also developed integrative research opportunities for extramural and intramural scientists through, respectively, the Disease Investigation for Specialized Clinically Oriented Ventures in Environmental Research (DISCOVER) and Director’s Challenge: DIR Program in Integrative Research. These opportunities, conceptually derived from the Research Teams of the Future Initiative of the NIH Roadmap, will enhance the interaction between basic and applied investigators by focusing on environmentally mediated diseases. Additionally, we have reengineered our environmental health sciences core research centers to focus on human health, and appropriated funds and developed plans to build an outpatient clinical research unit at our intramural research facility in Research Triangle Park, North Carolina.

In the area of training, we recognize the need to train MDs and PhDs in clinical research, and are developing interdisciplinary approaches to training for our predoctoral and postdoctoral students. We recognize the need to move trainees from mentored to independent research, and are strong supporters of the NIH Pathway to Independence Award. We have also established the Outstanding New Environmental Scientist (ONES) program to support the development of independent investigators and recruit talented emerging scientists to our field.

Another critical area is the need to develop sensors and biomarkers that provide precision in the personalized assessment of environmental exposures. One way this will be accomplished is through our involvement in the new NIH Genes and Environment Initiative, a joint research effort to combine genetic analysis and environmental technology to understand the causes of common diseases.

In an era of limited resources, a distinct challenge for the leadership of the NIEHS will be to calculate appropriate funding priorities to best achieve our goals of creating and supporting interdisciplinary and integrated research teams while encouraging and rewarding independent, investigator-initiated science. The combination of these two approaches will ensure that the most creative and innovative minds and ideas are focused on our emerging scientific issues. Recognizing that our greatest asset is our investigators, we will prioritize our trainees, emerging investigators, and established independent investigators when considering our funding options. After addressing these fundamental priorities, we will then direct funding to programs and infrastructure that support our vision.

These are challenging times for scientific research, but the NIEHS is moving forward with an aggressive Strategic Plan to enhance the opportunities within environmental health to bring the field’s unique strengths to bear on understanding the causes and development of complex human diseases. It is my hope that this Strategic Plan will set the NIEHS off on a voyage of discovery and success. I invite you along for what promises to be an exciting ride.

## Figures and Tables

**Figure:**
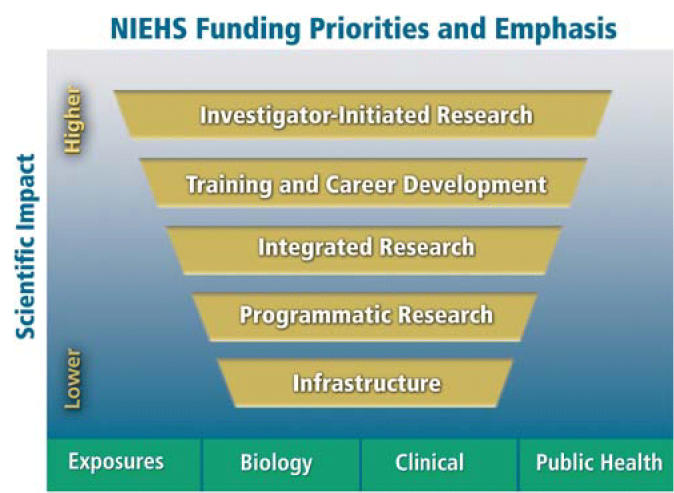


**Figure:**